# Electrostatic force microscopy for the accurate characterization of interphases in nanocomposites

**DOI:** 10.3762/bjnano.9.279

**Published:** 2018-12-07

**Authors:** Diana El Khoury, Richard Arinero, Jean-Charles Laurentie, Mikhaël Bechelany, Michel Ramonda, Jérôme Castellon

**Affiliations:** 1 Institut d’Électronique et des Systèmes, Université de Montpellier, Montpellier, France; 2 Institut Européen des Membranes, IEM - UMR 5635, ENSCM, CNRS, Montpellier, France; 3 Centre de technologie de Montpellier, Université de Montpellier, Montpellier, France

**Keywords:** atomic force microscopy, building-block materials, dielectric permittivity, electrostatic force microscopy, finite element simulation, interphases, nanocomposites

## Abstract

The unusual properties of nanocomposites are commonly explained by the structure of their interphase. Therefore, these nanoscale interphase regions need to be precisely characterized; however, the existing high resolution experimental methods have not been reliably adapted to this purpose. Electrostatic force microscopy (EFM) represents a promising technique to fulfill this objective, although no complete and accurate interphase study has been published to date and EFM signal interpretation is not straightforward. The aim of this work was to establish accurate EFM signal analysis methods to investigate interphases in nanodielectrics using three experimental protocols. Samples with well-known, controllable properties were designed and synthesized to electrostatically model nanodielectrics with the aim of “calibrating” the EFM technique for future interphase studies. EFM was demonstrated to be able to discriminate between alumina and silicon dioxide interphase layers of 50 and 100 nm thickness deposited over polystyrene spheres and different types of matrix materials. Consistent permittivity values were also deduced by comparison of experimental data and numerical simulations, as well as the interface state of silicone dioxide layers.

## Introduction

Composite nanomaterials (often referred to as “nanodielectrics” by the dielectrics community) can be synthesized by including dielectric nanoparticles in a polymeric matrix and are often used as insulating material [1–3]. Although the mechanical and thermal behavior of the base insulating polymer can be enhanced by microcomposites, its electrical performance is usually degraded [4–5]. On the other hand, the incorporation of nanofillers (1–10 wt %) into these polymers improves the dielectric properties of the resulting material, while meeting the thermal, mechanical and cost requirements [6–9].

This unusual behavior of nanocomposites is due to the interfacial region between the nanoparticles and polymer, also called the “interphase”. The interphase region can range from a few angstroms to a few nanometers, and usually has properties that differ from those of the polymer and filler. For instance, although the inorganic filler usually displays higher permittivity values than the polymer, the resulting nanofiller mixture in the matrix presents, at low nanofiller concentrations, a surprisingly lower dielectric constant than that of the two mixture components [10–13]. It is commonly agreed that the surface interaction of the nanoinclusions with the host polymer acts to rearrange the polymer chains and reduces their mobility at the interface [12–14]. The mobility reduction can lead to a decrease of the permittivity of the polar polymer, and consequently, also of the interphase. Moreover, it has been reported that water can be absorbed at the interface [15–17]. Water molecules within the interphase increase its effective dielectric constant, and this can explain the unexpectedly higher nanocomposite permittivity. Therefore, the local characterization of the interphase is of utmost importance to explain most of the nanocomposite macroscopic behavior, particularly their electrical polarization properties.

This local characterization requires high-resolution techniques that are sensitive to the dielectric properties of the material. These two conditions are fulfilled by electrostatic force microscopy (EFM) [18–19]. EFM is an atomic force microscopy (AFM)-based electrostatic method in which a conductive tip and a metallic sample holder are used. The probe-to-stage system is electrically polarized for the detection of electrostatic forces or force gradients. A proper interpretation of EFM results allows for the determination of the dielectric permittivity and dimensions of the sample components. Importantly, EFM is particularly suitable for electrical insulators, as opposed to electron microscopy where the rough specimen preparation procedures and electron beam bombardment can irreversibly electrically charge the material [20–21]. Moreover, while in the case of composites with 1D or 2D inclusions, the interphase can be directly accessed after cross-sectioning [20–21]; interphase characterization is more difficult for nanoparticle-filled materials. Indeed, the interphase is likely to be confined between the particles and the matrix below the probed region. Therefore, as the probing field is electrical in EFM, and this technology is expected to offer higher subsurface sensitivity compared with other scanning probe microscopy methods [22–25].

Subsurface imaging and 3D-multilayered structure studies with EFM have resulted in advances in our knowledge of specific types of materials. For instance, EFM comparison of the nanofiller diameter before and after insertion into the polymer matrix [26] showed an increase of the apparent particle diameter in the matrix that was attributed to the interphase. However, some experimental conditions during the comparison were not similar. Moreover, the highly probable presence of a matrix layer over the particles near the surface in the nanocomposite was ignored, although a matrix covering the nanoparticles would increase their apparent size even without an interphase. In a more rigorous study [27], Peng et al. detected, in sliced specimens of a nanodielectric, unexpectedly lower EFM signals above the filled matrix regions compared with the supposedly unfilled regions. To explain this EFM signal reduction, the authors hypothesized that an interphase with lower permittivity than that of particles and fillers surrounds the particles. However, the authors did not compare this remarkable change in local dielectric permittivity with macroscopic dielectric spectroscopy measurements. Nevertheless, the interphase characterization in nanocomposites is still inadequate. One common issue in these EFM-based works is that the exact source of the measured signal was not completely identified. Therefore, the measurement of complex materials remains a big challenge, mainly due to the complex geometry of the probe that scatters the electric field, and the long range nature of the electrostatic forces that complicate the identification of the actual probed region.

Therefore, the objectives of this study were to determine whether EFM can identify an interface region, and most importantly, to identify the appropriate experimental methods to extract the artifact-free EFM signal of the interphase. To this aim, materials of relatively known and modifiable composition and shape that electrostatically mimic a nanodielectric were designed and prepared to “calibrate” the technique. These were made of a stack of three dielectric constituents that represent a simplified configuration of the particle–interphase–matrix assembly found in nanodielectrics. Specifically, polystyrene (PS) nanoparticles of 380 nm diameter were prepared by self-assembly on metallized substrates. Then, two shells of variable thicknesses (50 and 100 nm) were deposited or grown over the whole sample surface. Aluminum oxide (Al_2_O_3_) shells were prepared using the atomic layer deposition (ALD) method, polyvinyl acetate (PVAc) shells by spin coating, and silicon dioxide (SiO_2_) shells by plasma sputtering deposition (PSD). The signature of each dielectric constituent was correlated to its dielectric permittivity. EFM measurements were performed using the double-pass method, while extracting the frequency shifts due to the acting electrostatic force gradients over the probe. During the second scan, the system was polarized at an AC voltage and the tip was retracted from the surface at a known distance, called the lift distance. At this stage, the component of the frequency shift that varies at the double frequency of the applied electrical voltage was extracted because it represents the purely capacitive response of the material. Finally, the experimental results were compared to finite element numerical simulations, obtained with the AC–DC module, electrostatics physics interface, of the Comsol^®^ Multiphysics software.

## Results and Discussion

In our previous work, we verified that EFM can distinguish homogeneous from heterogeneous stacked materials and provide information on the permittivity of their constituents relative to each other [28]. For instance, in materials made of particles with only one shell, the shell permittivity (ε_i_) relative to that of the particle (ε_p_) can be best obtained from signal comparisons at the center of the particles. Particularly, at a constant tip–sample distance, the signal increases with the thickness of the added material, and this can only be explained by an increase of the effective permittivity of the global material. This confirms the higher permittivity of the additional component compared with the initial one. Based on these findings, we developed three experimental approaches in which different model samples were compared to detect and characterize the interphase in a nanodielectric model composed of particle + interphase + matrix.

In all the tested model samples, the particle topography could be determined by a line scan because the covering layers were prepared in such a way to keep the curvature associated with the spheres. Completely embedded particles could also be used. However, the configuration where the particle protrudes from the surface has been described in previous works on interphases in “real” nanocomposite systems, using mechanical scanning probe microscopy techniques [29–31], and EFM [26–27,32–33]. Moreover, discriminating the particles from the topography is important in the case of nanocomposites that include particles and matrix with low dielectric permittivity difference.

### Approach 1: PS + Al_2_O_3_ and PS + Al_2_O_3_ + PVAc

**Figure 1:**
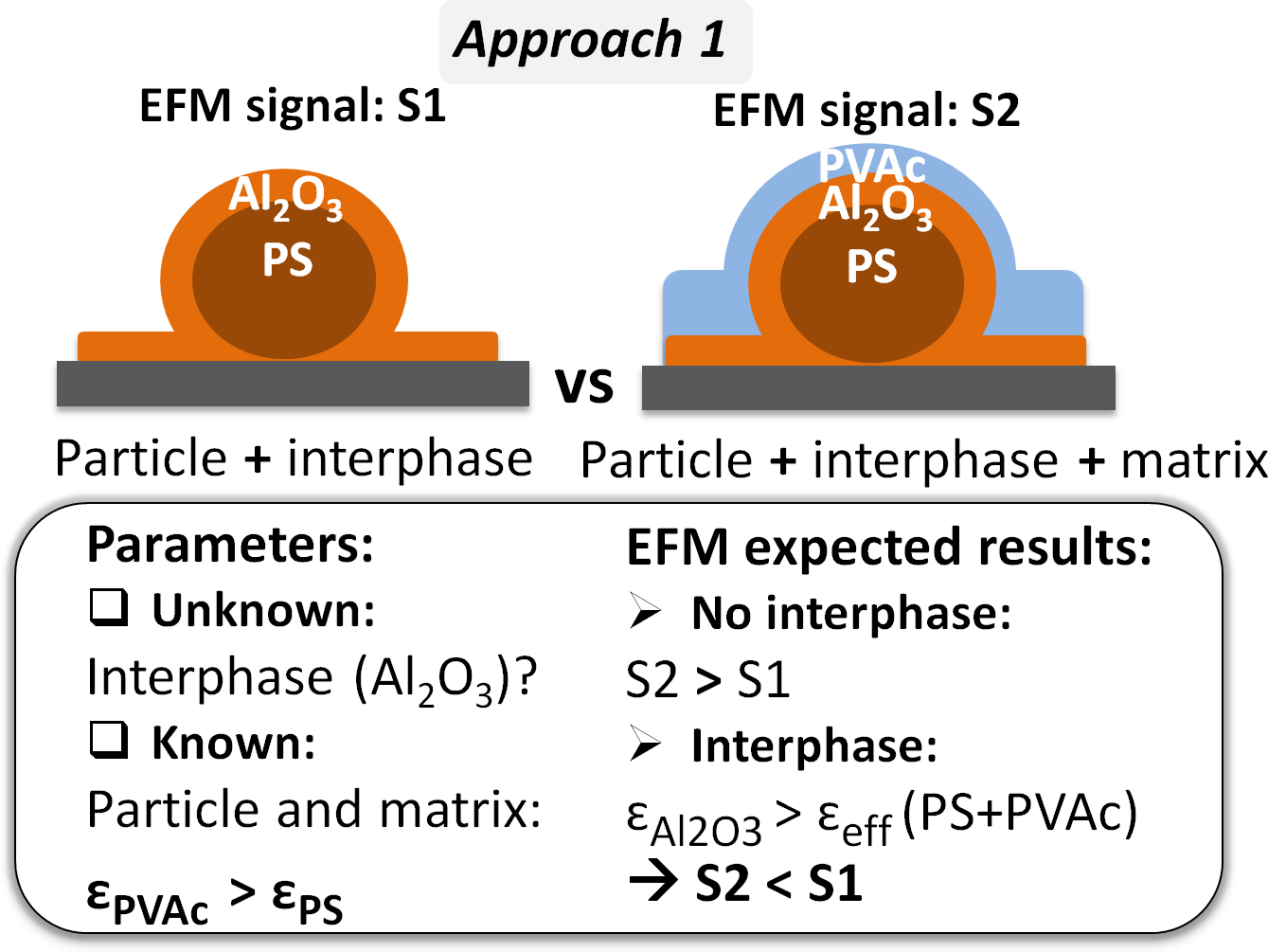
Interphase detection, approach 1: schematic representation of the samples to be compared, and methodology.

In the first method, the EFM signals for PS + 100 nm Al_2_O_3_ and PS + 100 nm Al_2_O_3_ + PVAc samples were compared (Figure 1). These samples mimicked a nanodielectric that has interphase (Al_2_O_3_) without and with a matrix surface layer, respectively.

Al_2_O_3_ was the interphase model material to be detected, while PVAc (matrix) and PS (particle) were the known materials (depicted in Figure 1). As in the absence of interphase, ε_PVAc_ = 3.2 > ε_PS_ = 2.6, PVAc addition over the particles should increase the signal [34–36]. Conversely, in the presence of the Al_2_O_3_ interphase, alumina is supposed to enhance the effective permittivity of the covered particles and, consequently, PVAc addition should decrease the signal (i.e., interfacial effect due to the presence of a layer above the particles with higher permittivity compared with the PS + PVAc assembly). As changes in sample dimensions and permittivity values should be relatively small, the statistical analysis was performed using EFM measurements acquired on 12 × 3 µm^2^ images based on an average of 30 particles [37].

**Figure 2:**
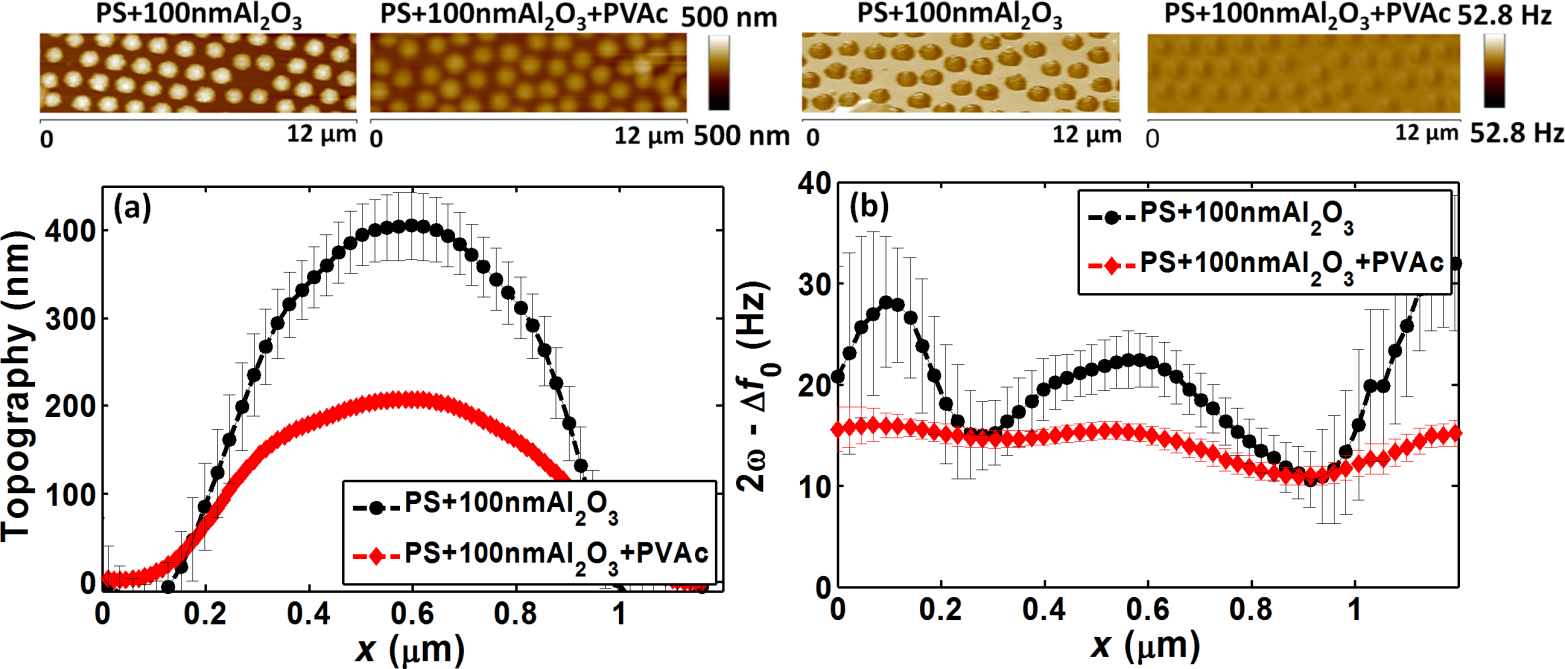
Approach 1 (PS + 100 nm Al_2_O_3_ and PS + 100 nm Al_2_O_3_ + PVAc samples): (a) average topography profiles, (b) average EFM signal profiles, and the corresponding AFM and EFM images (upper panels); tip–sample distance *z* ≈ 21 nm; reproduced with permission from [39], copyright 2018 IEEE.

The calculated average topography and electrical frequency shift (2ω − Δ*f*_0_) profiles for PS + 100 nm Al_2_O_3_ and PS + 100 nm Al_2_O_3_ + PVAc samples are presented in Figure 2a and 2b. The topography height was lower in samples with than in those without the matrix surface layer. Additional nanomechanical measurements indicated that this decrease was due to the non-uniformly thick spin-coated PVAc film [38]. In fact, as our samples were highly rough owing to the sub-micrometer PS spheres, spin-coating could not produce uniformly thin films [37]. However, this did not affect our study because the EFM results were compared at the center of the particles.

Concerning the EFM signals, the average values calculated around the center were 20.6 ± 1.9 Hz for samples without matrix and 14.6 ± 1 Hz for samples with PVAc. This indicated that the matrix decreased the overall central signal by ≈30%, supporting our hypothesis on the effect of the Al_2_O_3_ interphase layer. This result confirmed the sensitivity of the first approach within the correct geometry and permittivity ratios in order to detect the region at the interface between PS particles and PVAc matrix. However, this methodology is limited by its concept based on comparing nanodielectrics with and without an upper matrix layer. Indeed, it is difficult to produce samples that lack a surface matrix layer without influencing the initial properties of the interphase. Therefore, it would be more realistic to compare samples that model nanodielectrics with and without an interphase.

### Approach 2: PS + 50 nm SiO_2_ and PS + 100 nm Al_2_O_3_ + 50 nm SiO_2_

**Figure 3:**
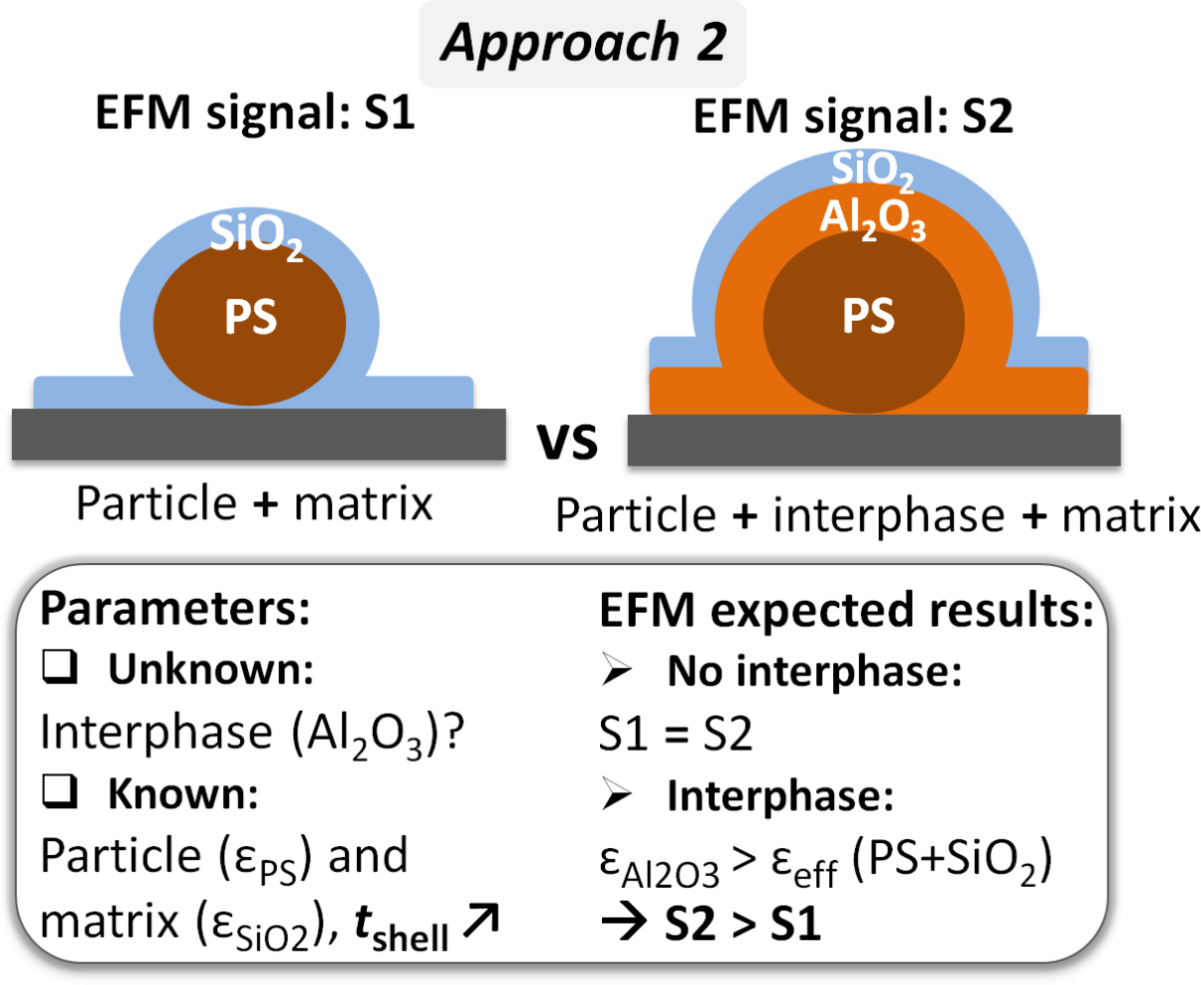
Interphase detection, approach 2: schematic representation of the samples to be compared, and methodology.

In the second approach, the comparison was between model nanodielectric samples without (PS + 50 nm SiO_2_) and with an interphase (PS + 100 nm Al_2_O_3_ + 50 nm SiO_2_) in which the matrix thickness was kept constant (Figure 3). To this aim, matrix deposition required a technique that allows the precise control of the thickness. For this reason, SiO_2_ was used instead of PVAc because it can be deposited by PSD. This method can precisely and homogeneously spread SiO_2_ molecules over the whole sample surface, quite similar to ALD, as explained in the Experimental section [40].

In approach 2 (Figure 3), the signal between the compared samples should be similar in the absence of an interphase, whereas the Al_2_O_3_ interphase is expected to strengthen the signal, confirming the contribution of an additional layer with a higher permittivity than that of the PS + SiO_2_ sample. Indeed, as deduced from [28] and mentioned earlier, when the EFM signal increases in the presence of a thicker layer at the same tip–sample distance, this indicates that the added material possesses a dielectric permittivity higher than that of the initial particle + shell assembly. Although in our comparisons the added material was not at the surface but in the middle, the principle was the same [38].

**Figure 4:**
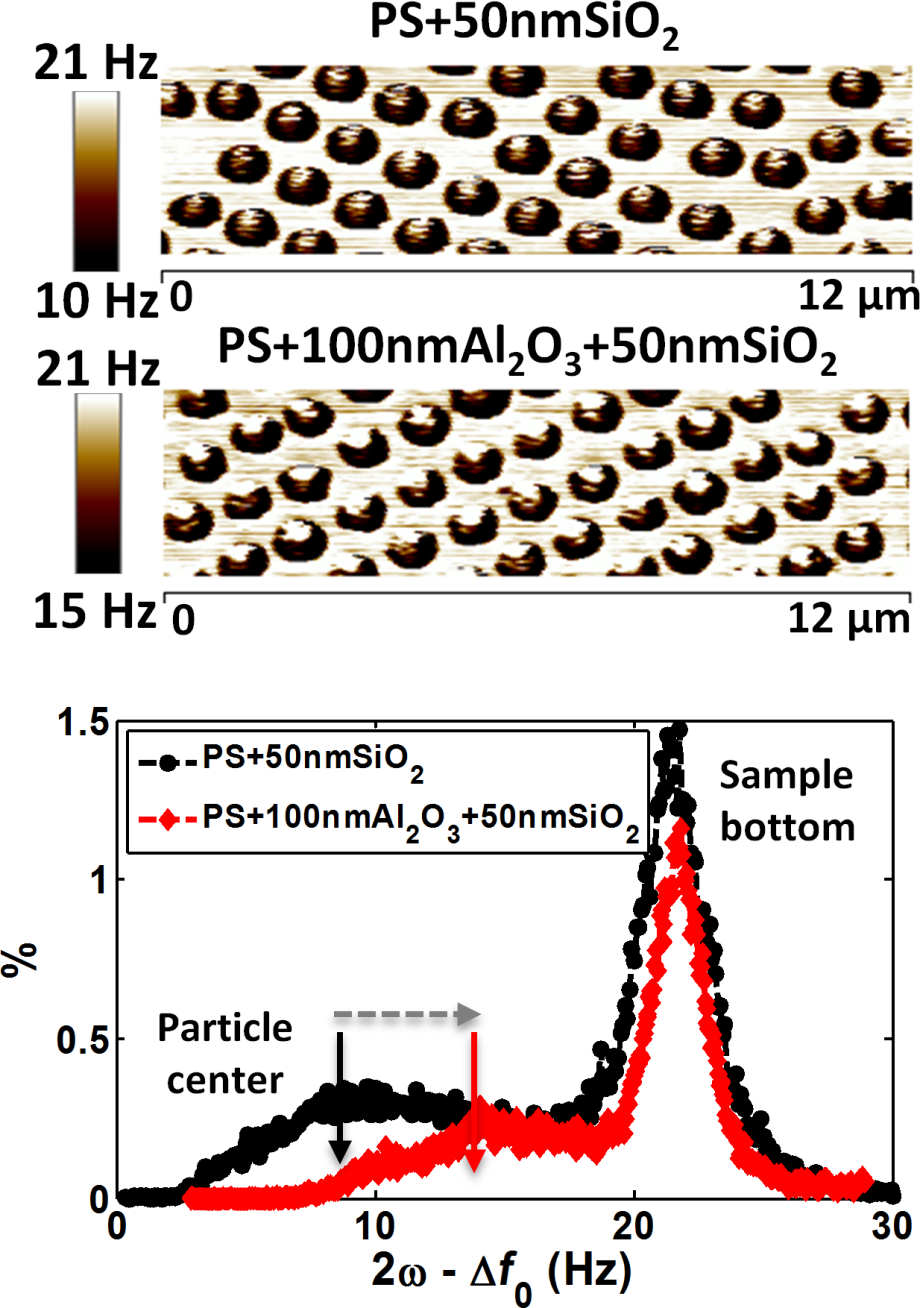
Approach 2: EFM images of PS + 50nmSiO_2_ (upper) and PS + 100 nm Al_2_O_3_ + 50 nm SiO_2_ (middle panel) samples, and their corresponding EFM signal distribution histogram (bottom); *z* = 21 nm.

A histogram illustrating the EFM signal distribution of PS + 50 nm SiO_2_ and PS + 100 nm Al_2_O_3_ + 50 nm SiO_2_ samples (Figure 4) showed the presence of two peaks. The maximum corresponded to the response from the bottom of the sample, and the lowest peak was the response from the particle center. A shift towards higher *∆f*_0_ was observed for samples with the additional intermediate Al_2_O_3_ layer (arrow). This indicates that EFM can deduce the presence of an embedded Al_2_O_3_ interphase layer underneath the SiO_2_ matrix, and corroborates the hypothesis underlying this approach.

This second approach is suitable to define the interphase relative dielectric permittivity, but only by comparison with that of the particle + matrix assembly. It can thus only indicate whether the interphase permittivity is higher or lower than the relative permittivity of the material with the highest permittivity (particle or matrix). Although such results are useful, the direct identification of the interphase, relative only to the matrix, is also essential. To this aim, a third method was evaluated.

### Approach 3: comparisons of samples with similar dimensions

**Figure 5:**
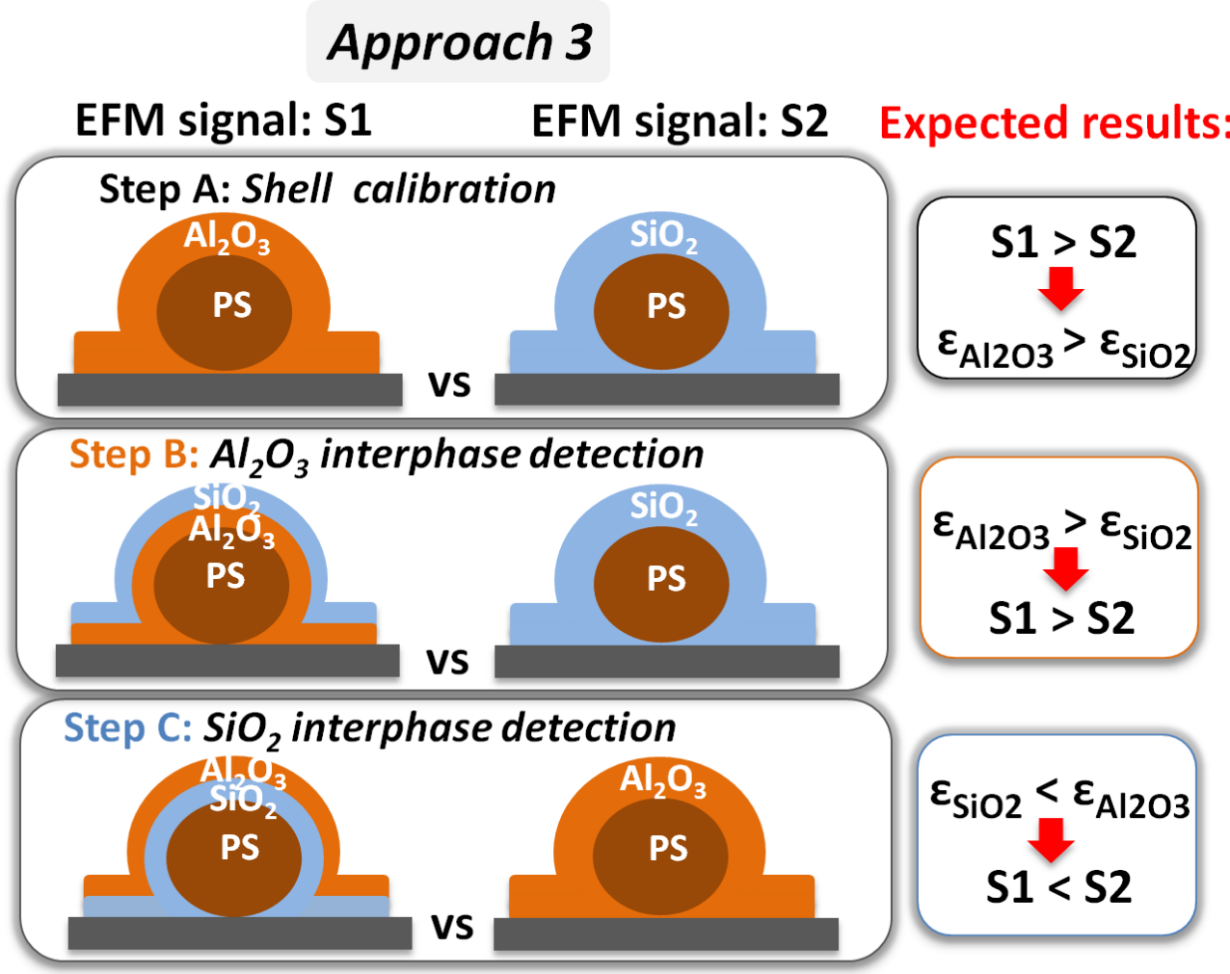
The three steps of approach 3: compared samples and methodologies.

Like for approach 2, in approach 3, the comparison was between samples that model nanodielectrics with and without an interphase and in which the total shell thickness was kept constant. This removed any confusion due to the sample topography, and consequently the relative permittivity of the shell material became the prominent parameter. For this reason, approach 3 was divided in three steps outlined in Figure 5. In step A, the permittivity of the studied shells (Al_2_O_3_ and SiO_2_) was calibrated. In steps B and C, the Al_2_O_3_ interphase underneath (B) and covering (C) the SiO_2_ matrix was detected. If these materials behave normally, this approach should allow Al_2_O_3_ to be distinguished from SiO_2_ on the basis of the EFM signal increase, when it replaces it.

A tip–sample distance *z* of around 30.1 nm was kept for all measurements. The result reproducibility was always verified at several sample regions, and most of the time (in several comparable samples) measured with different probes. Since, as in this section, experimental data and simulations for a large set of samples were compared, only the results obtained with a specific probe will be presented.

### Tip calibration

**Figure 6:**
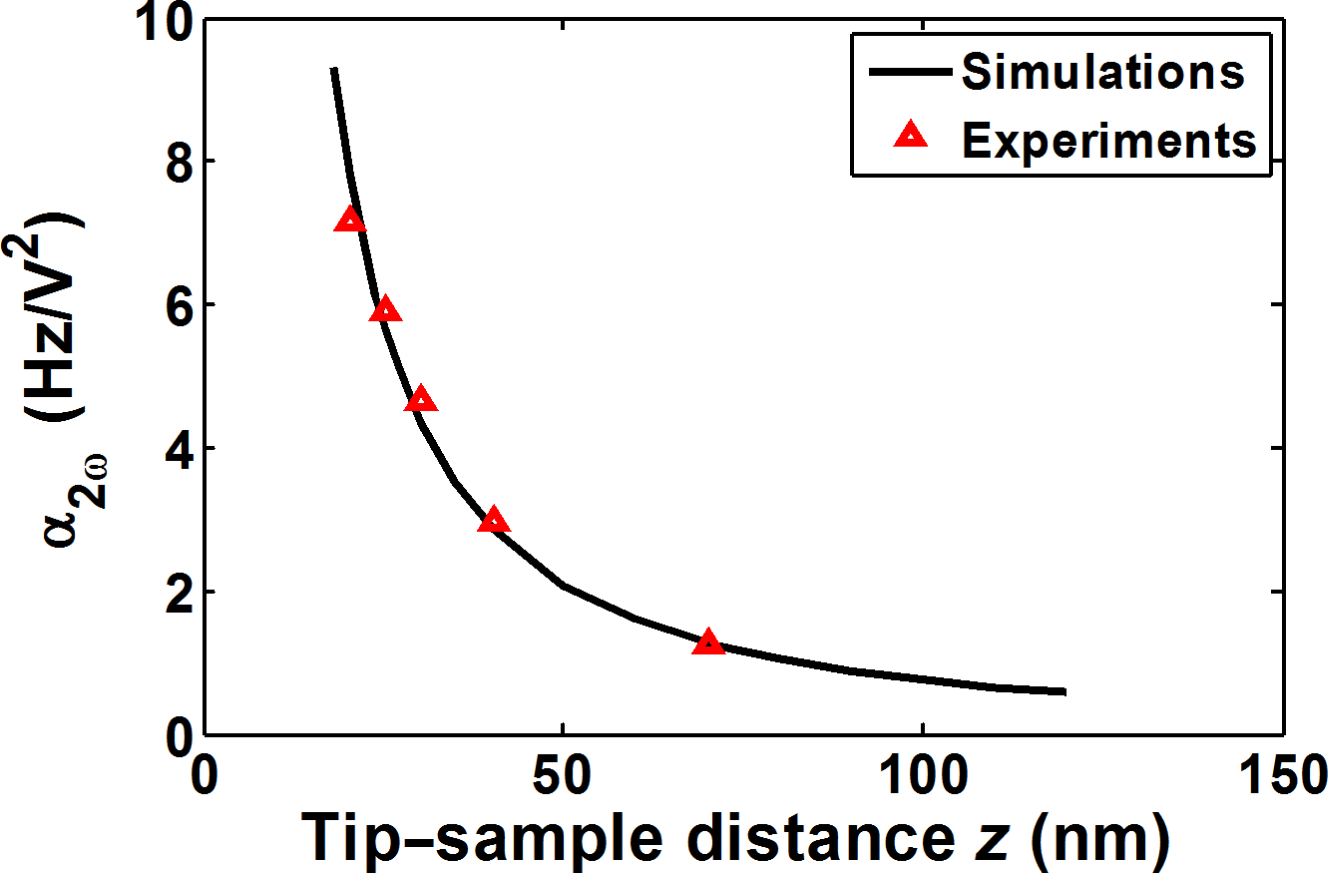
Tip calibration using the signal vs *z* curve. Experimental data fit simulations (tip radius of 29 nm and cone half-angle of 15º radius) with a total error of 4.6%.

The first step to compare experimental data and simulations was to calibrate the tip geometry. One-point curves were performed using a metallic small-sized sample (10 nm × 2.5 nm), and the parabolic coefficient α_2ω_ (Hz/V^2^) was calculated (please refer to Equation 1 in Experimental section). A tip radius of 28 nm and cone half-angle of 15º best fitted the experimental results, as indicated by the 4.6% total error compared with the simulations (Figure 6). Errors were measured as follows:

[2] $$\text{error}=100\times \left|\frac{\alpha_{\text{exp}}-\alpha_{\text{sim}}}{\alpha_{\text{exp}}}\right|$$

### Reference polystyrene sample

**Figure 7:**
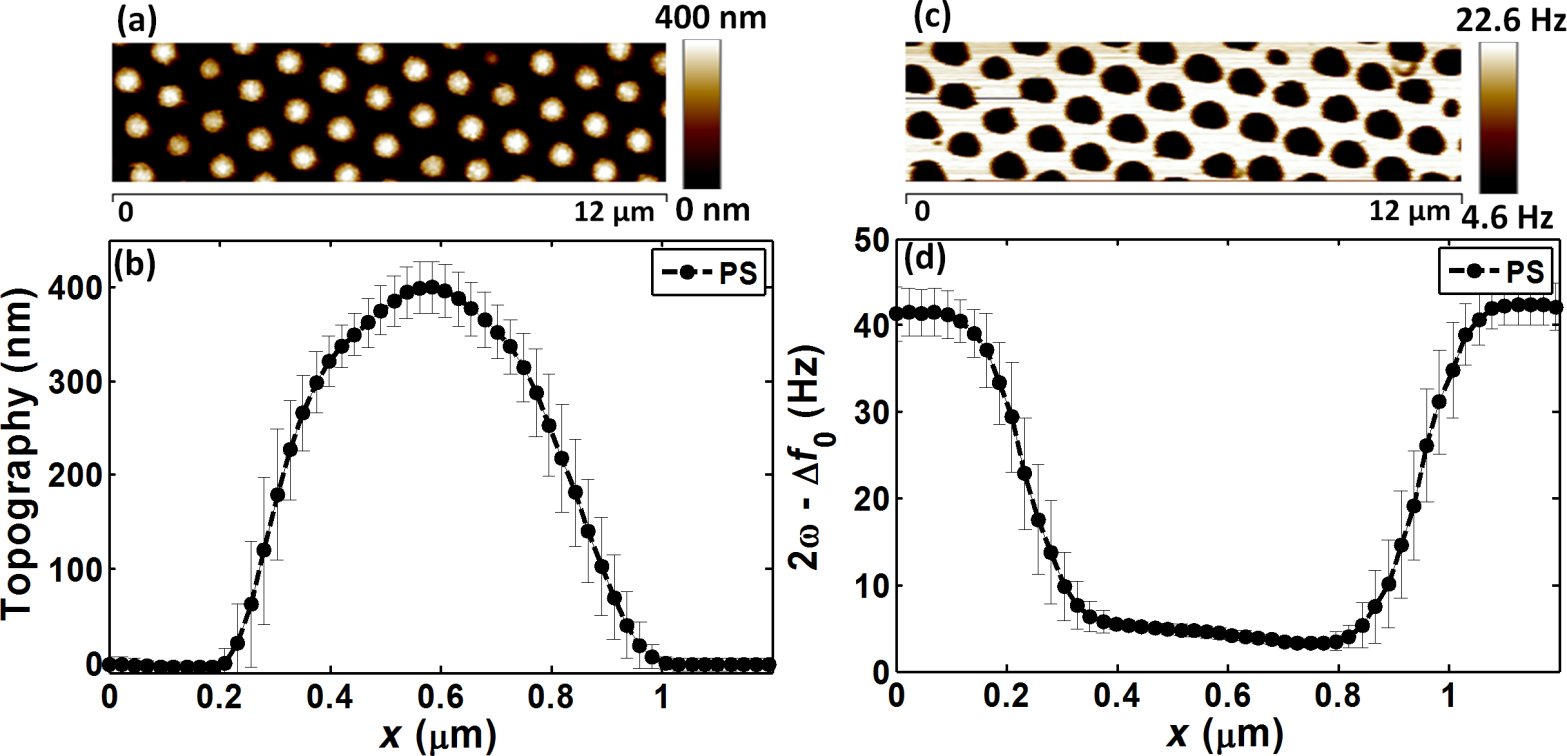
PS calibration – average cross-sectional profiles measured using a typical reference PS sample. (a) AFM image and (b) topography; (c) EFM image and (d) EFM response.

The EFM and AFM images of the reference PS sample (Figure 7a,c) were used to extract the average topography and EFM cross-sectional profiles (Figure 7b,d). The average value of both profiles around the sample center was also calculated. The PS mean height was 383 ± 29 nm and the corresponding α_2ω_ was 0.49 ± 0.07 Hz/V^2^. By implementing this PS geometry and experimental parameters in our numerical model, a PS dielectric permittivity of 2.6 was obtained that fit with the experimental data (0.08% error) (Table 1).

**Table 1:** Experimental data and simulations for a typical reference polystyrene sample, using *z* = 30.14 nm, tip-calibration parameters (28 nm, 15°), and average particle diameter = 382 nm. A particle permittivity of 2.6 fits simulations with 0.8% error.

	Experiment	Simulation		Comparison
	α_2ω_ (Hz/V^2^)	α_2ω_ (Hz/V^2^)	ε	Error (%)

Polystyrene	0.49 ± 0.07	0.49	2.60	0.80

### Step A: PS + 100 nm Al_2_O_3_ and PS + 100 nm SiO_2_ (shell calibration)

**Figure 8:**
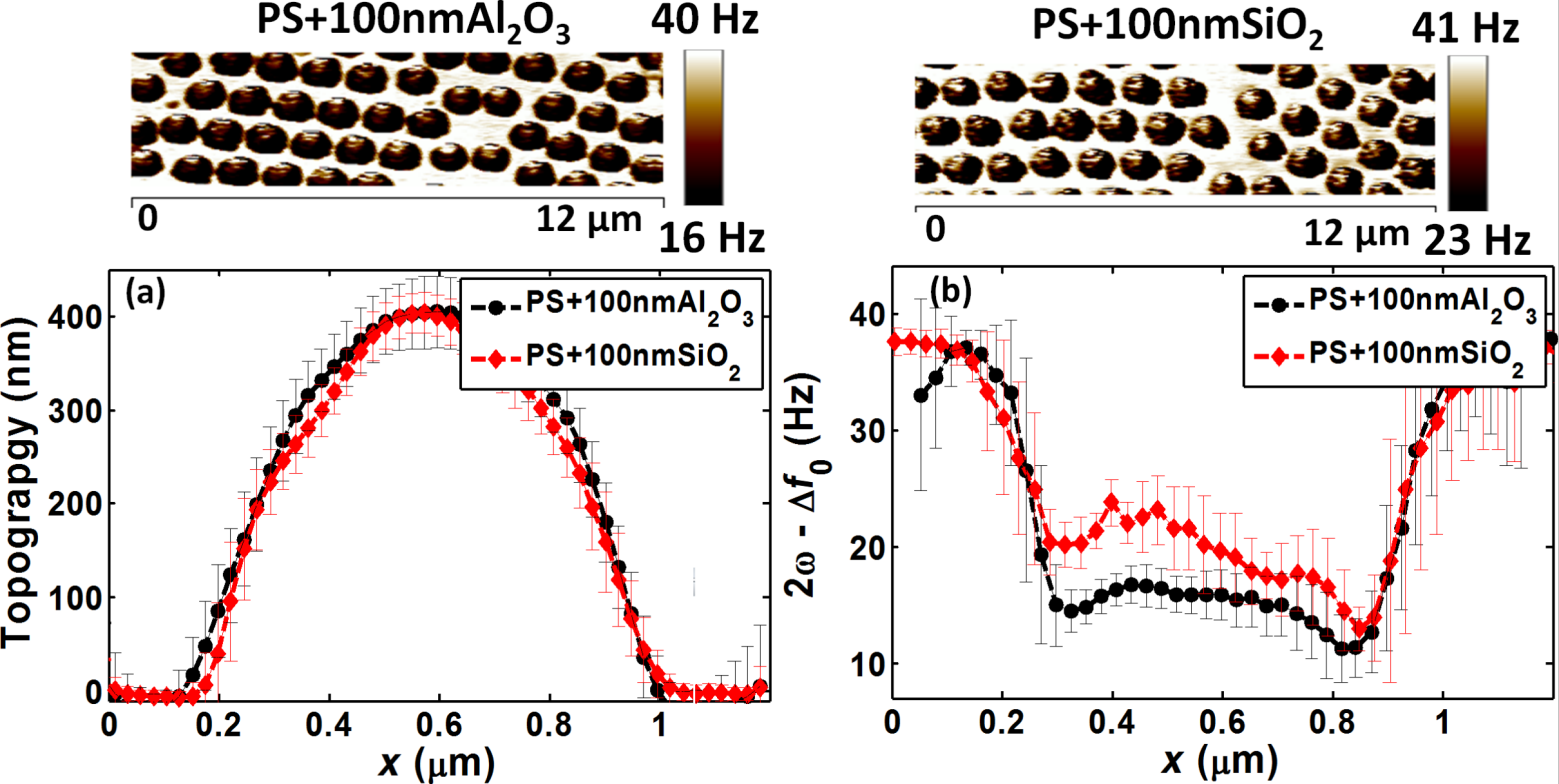
Approach 3 (step A) PS + 100 nm Al_2_O_3_ and PS + 100 nm SiO_2_ samples: (a) Average topography profiles, (b) average EFM signal profiles, and their corresponding EFM images (upper panels).

In step A (Figure 5 – shell calibration), the average topography profiles obtained by AFM image analysis (Figure 8a) between PS + 100 nm Al_2_O_3_ and PS + 100 nm SiO_2_ samples were comparable, as expected from the calibrated preparation process. Conversely, the average EFM profiles of the same samples (Figure 8b), also presented in the images of Figure 8 upper panel, were different in terms of signal magnitude, at the center of the particles and also at the bottom regions. As our samples had similar thicknesses, this difference could be explained by the different SiO_2_ and Al_2_O_3_ dielectric permittivity values. However, surprisingly, the EFM signal was higher with SiO_2_ than with Al_2_O_3_, although the SiO_2_ permittivity is lower than that of Al_2_O_3_ (3.9 for SiO_2_ and 9.8 for Al_2_O_3_) [34,36,41]. As Al_2_O_3_ shells have already been well characterized in the previous sections and also in [28] and showed predictable performance, this abnormal dielectric response could be attributed to SiO_2_.

In the subsequent step, we correlated the experimental results with the simulations to allow for the calculation of the apparent permittivity of the shells, particularly SiO_2_. PS was modeled with a permittivity of 2.6, deduced during the calibration process (Table 1). For Al_2_O_3_, the dielectric permittivity of 9.8 was in accordance with the experimental data (error = 3.2%), and also in agreement with the expected Al_2_O_3_ behavior. Errors below 5% are widely acceptable, considering the errors related to tip and sphere calibration, film thicknesses, and the inherent experimental errors. The agreement between experimental data and simulations may also indicate that there is no additional interfacial effect between the particle and the shell, or at least, no dominant effect at this level of the material dimensions and properties. On the other hand, for SiO_2_, an apparent permittivity of ε_app_ = 17 best fits the experimental data with an error of 0.6%. This permittivity was much higher than the usual SiO_2_ permittivity value (around 3.9). Consequently, step B and C measurements were performed by taking into account these values.

### Step B: PS + 50 nm Al_2_O_3_ + 50 nm SiO_2_ and PS + 100 nm SiO_2_ (Al_2_O_3_ interphase detection)

After the validation of the deposition of similarly thick layers by ALD (Al_2_O_3_) and PSD (SiO_2_) and the calibration of the SiO_2_ and Al_2_O_3_ layers, stacking layers of SiO_2_ or Al_2_O_3_ over PS for detecting the intermediate material becomes relevant. The second main step aimed for addressing the interphase detection by comparing the EFM response of PS + 50 nm Al_2_O_3_ + 50 nm SiO_2_ and PS + 100 nm SiO_2_ samples. In this model, the interphase layer was 50 nm Al_2_O_3_ (Figure 5, step B).

**Figure 9:**
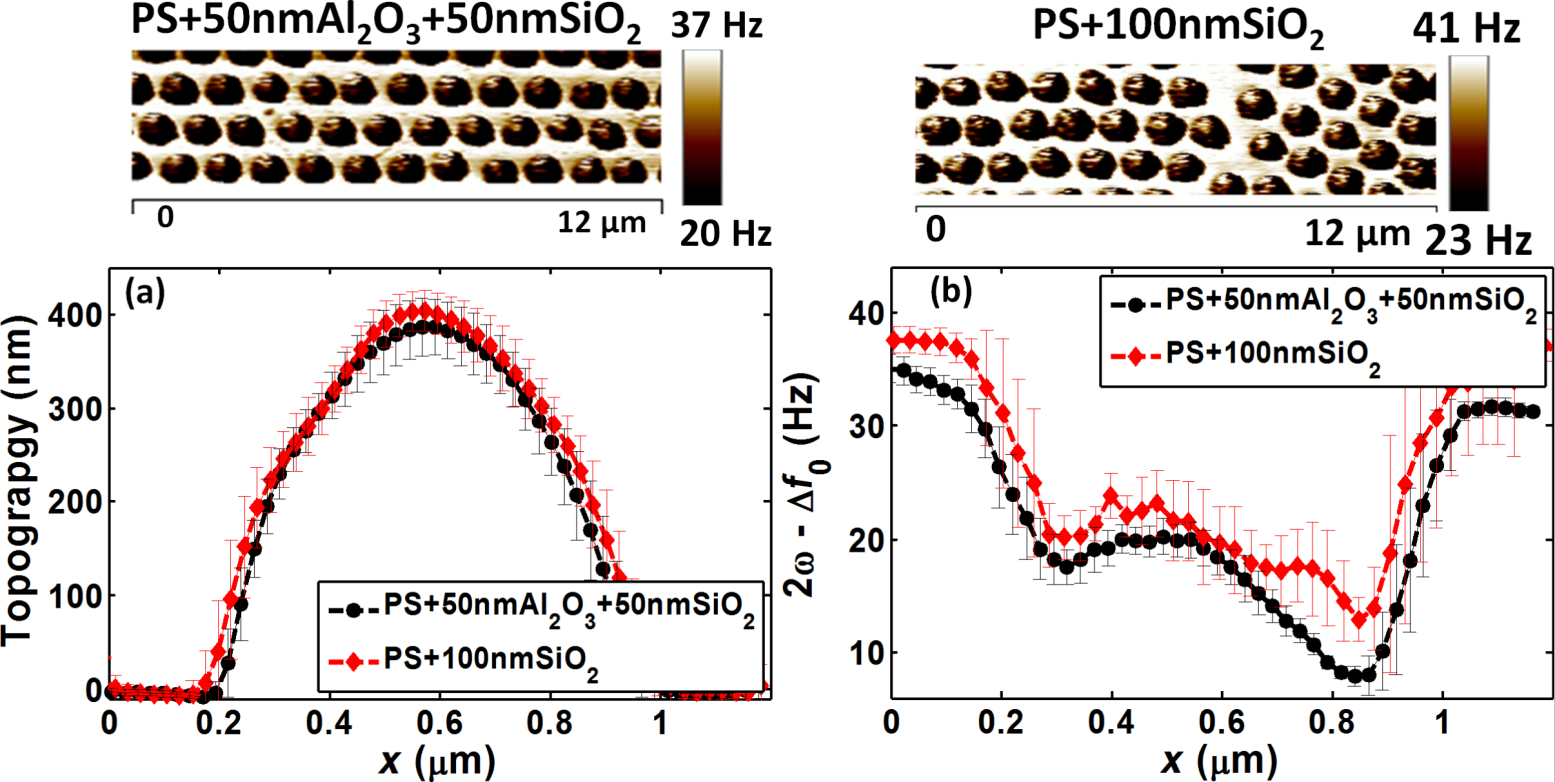
Approach 3 (step B) PS + 50 nm Al_2_O_3_ + 50 nm SiO_2_ and PS + 100 nm SiO_2_ samples: (a) Average topography profiles, (b) average EFM signal profiles (lower panel) and the corresponding EFM images (upper panels). To avoid border effects, the EFM cross-sections were collected slightly far from the center and this is reflected by the change of the apparent particle width on the EFM average profiles.

Figure 9 shows that the average topography profiles of PS + 50 nm Al_2_O_3_ + 50 nm SiO_2_ and PS + 100 nm SiO_2_ samples were similar. Conversely, analysis of the EFM average response of Figure 9 upper panels indicated that the signal around the center decreased in samples with an intermediate Al_2_O_3_ layer (Figure 9b), as shown also by comparison of the mean values around the center (Table 2). This indicated the presence of an interphase effect. Moreover, the signal decrease with the Al_2_O_3_ interphase confirmed that its apparent permittivity was lower than that of SiO_2_. The abnormal behavior of these two materials was in accordance with the results obtained in step A. In fact, when simulations were performed using permittivity values of 9.8 and 17 for Al_2_O_3_ and SiO_2_, respectively, the simulations matched the experimental data (see Table 2).

**Table 2:** Experimental data and simulations for PS + 50 nm Al_2_O_3_ + 50 nm SiO_2_ and PS + 100 nm SiO_2_. The Al_2_O_3_ subsurface layer had a dielectric permittivity value of 9.8 (error = 5%) for a calibrated SiO_2_ upper layer with an apparent permittivity value of 17 (step A).

	Signal			Permittivity	
	Exp. (Hz/V^2^)	Sim. (Hz/V^2^)	Error (%)	ε_interphase_	ε_matrix_

PS + 100 nm SiO_2_	2.29 ± 0.33	2.27	0.60	ε_SiO2_ = 17	ε_SiO2_ = 17
PS + 50 nm Al_2_O_3_ + 50 nm SiO_2_	2.01 ± 0.18	2.11	5.10	ε_Al2O3_ = 9.8	ε_SiO2_ = 17

The relatively small difference between PS + 50 nm Al_2_O_3_ + 50 nm SiO_2_ and PS + 100 nm SiO_2_ signals can be explained by the thickness of the upper layer (50 nm) that limits the electric field penetration, and consequently, the effect of the subsurface layer on the signal [38,42]. Moreover, the high dielectric polarization response of the surface material also masks the response from the deeper parts. Besides, the difference between the deduced apparent permittivity of SiO_2_ (ε_app_ = 17) and of Al_2_O_3_ (ε_Al2O3_ = 9.8) was relatively low. Notably, high dielectric permittivity materials are known to be difficult to distinguish [43], even if their effective permittivity decreases when they are placed on a material with lower permittivity (ε_PS_ = 2.6 < 9.8 and 17). Moreover, the reduction in sample effective permittivity enhances the EFM sensitivity to the top film [38,44].

### Step C: PS + 50 nm SiO_2_ + 50 nm Al_2_O_3_ and PS + 100 nm Al_2_O_3_ (SiO_2_ interphase detection)

In step C, the comparison concerned the EFM response of PS + 50 nm SiO_2_ + 50 nm Al_2_O_3_ and PS + 100 nm Al_2_O_3_ samples (Figure 5, step C).

**Figure 10:**
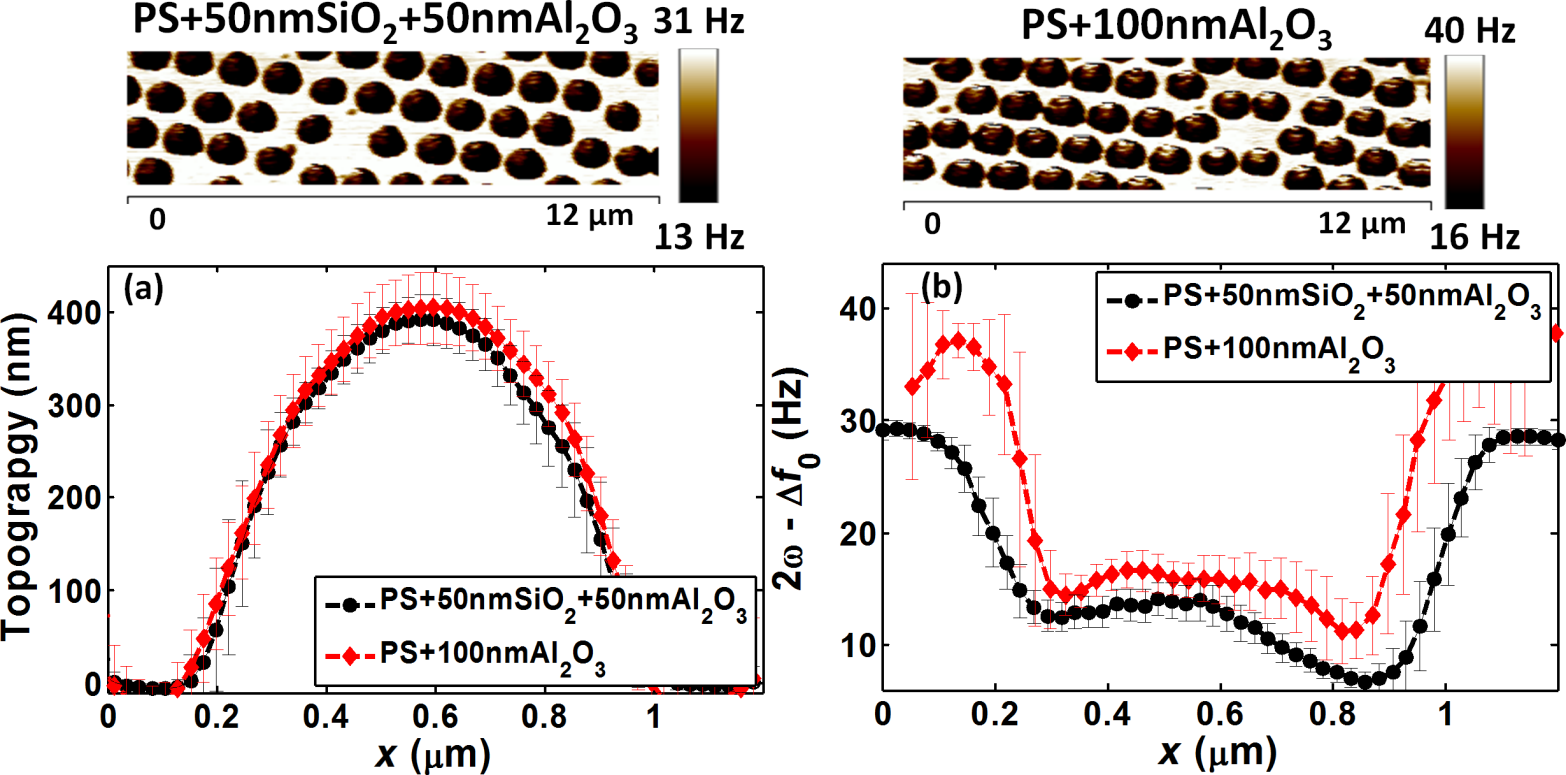
Approach 3 - Step C (PS + 50 nm SiO_2_ + 50 nm Al_2_O_3_ and PS + 100 nm Al_2_O_3_ samples). a) Average topography profiles, b) average EFM signal profiles (lower panel) and the corresponding EFM images (upper panels).

As observed in step B, while the average topography profiles were comparable (Figure 10a), the average EFM profiles were different between PS + 50 nm SiO_2_ + 50 nm Al_2_O_3_ and PS + 100 nm Al_2_O_3_ samples of Figure 10 upper panels (Figure 10b). The average electrical signal clearly decreased both at the particle center and at the bare substrate regions in PS + 50 nm SiO_2_ + 50 nm Al_2_O_3,_ where a layer of 50 nm SiO_2_ was added underneath the 50 nm Al_2_O_3_ layer, compared with PS + 100 nm Al_2_O_3_, where only a 100 nm Al_2_O_3_ layer covered the PS.

In steps A and B, the apparent dielectric permittivity of SiO_2_ was 17. If this permittivity were maintained also when SiO_2_ was used as interphase layer, the EFM signal for SiO_2_-filled samples should have been higher than that of PS + 100 nm Al_2_O_3_ samples. This was not observed in the experimental conditions (Figure 10b). Conversely, the experimental results can be understood when using the nominal intrinsic bulk permittivity of SiO_2_ (around 3.9, thus lower than Al_2_O_3_ permittivity: ε_Al2O3_ = 9.8). This hypothesis was verified by simulations in which the SiO_2_ and Al_2_O_3_ permittivity values were set at 3.9 and 9.8, respectively (Table 3).

**Table 3:** Experimental data and simulations for PS + 50 nm SiO_2_ + 50 nm Al_2_O_3_ and PS + 100 nm Al_2_O_3_ samples. The subsurface SiO_2_ layer displays a dielectric permittivity value of 3.9 (error = 0.3%).

	Signal			Permittivity	
	Exp. (Hz/V^2^)	Sim. (Hz/V^2^)	Error (%)	ε_interphase_	ε_matrix_

PS + 100 nm Al_2_O_3_	1.71 ± 0.26	1.65	3.24	ε_Al2O3_ = 9.8	ε_Al2O3_ = 9.8
PS + 50 nm SiO_2_ + 50 nm Al_2_O_3_	1.48 ± 0.17	1.48	0.27	ε_SiO2_ = 3.9	ε_Al2O3_ = 9.8

In conclusion, steps B and C of approach 3 confirmed that EFM can be employed to characterize interfacial layers with similar configurations as those of the samples under study. Moreover, these results highlighted variable behavior of SiO_2_.

### Discussion on approach 3

As in all three steps (A, B and C), SiO_2_ was deposited using the same technique, intrinsic material changes (bulk SiO_2_) cannot explain the apparent SiO_2_ permittivity variations among the samples. Our experimental data indicate that the SiO_2_ permittivity is higher than the standard value when SiO_2_ is placed on the sample surface, but not when it is used as an interphase. As the experiments were performed under ambient conditions, and SiO_2_ is a highly hydrophilic material, it can be hypothesized that a thin water layer was adsorbed on the surface. The high dielectric polarizability and permittivity of water films could explain the higher effective permittivity of superficial SiO_2_. Along the same lines, the normal value of the SiO_2_ interphase (PS + 50 nm SiO_2_ + 50 nm Al_2_O_3_) could be linked to the presence of an Al_2_O_3_ layer on the surface of SiO_2_. Al_2_O_3_ was prepared by ALD operated at low vacuum (0.001 mbar) at 80 °C and left to acquire temperature and vacuum stability for about 40 minutes before starting its deposition. In these thermodynamic conditions, water molecules might evaporate and the 40 min interval before deposition should have been enough to allow H_2_O desorption [45].

Although both SiO_2_ and Al_2_O_3_ are considered highly hydrophilic materials [15,46], SiO_2_ seems to retain water molecules on its surface more readily than Al_2_O_3_, as observed in Figure 8, and as confirmed by measurements performed in a highly humid environment [38]. Indeed, in the presence of high humidity, only scanning over alumina-covered samples was possible. All used probes were fixed to the surface and were consequently crushed or became contaminated, requiring a tip change. As a water meniscus created between the tip and the surface in air environment can cause the sticking of the tip to the surface at low distances, layers that adsorb more water molecules are supposed to be responsible for greater attraction forces. Consequently, a water meniscus can cause dynamic imaging instabilities [47], and an important water adsorption/absorption could explain the surface status difference between Al_2_O_3_ and SiO_2_.

The apparently higher SiO_2_ hydrophilicity could be attributed to the chemistry of the surface states resulting from the deposition method. ALD is a smooth chemical vapor deposition technique, expected to leave hydroxide OH groups on the surface. Conversely, PSD might induce a more homogeneous ionic surface state; O^−^ and O_2_^−^ could be typically introduced due to the small amount of reactive oxygen gas that is added to the neutral argon gas during sputtering [48].

This result was also verified for superimposed planar films, as deduced from the bottom regions of the sample (Figure 9b). In [38], we also tested these findings on entirely planar samples. Nevertheless, these hypotheses on the SiO_2_ surface emphasize the need for better investigations.

### Discussion on the application for “real” nanodielectrics

The ultimate aim of this work is to use the proposed methodologies for investigating interphases in real materials where the only known parameters are the mixture components. With real nanodielectrics, the first, obvious step will be to compare materials that show unpredictable properties at the macroscale, indicating the possibility of the presence of interfacial effects. To this aim, a simple approach, inspired by Peng et al. work [27], would be thinning the material into slices, starting with a thickness slightly larger than the particle diameter. At this stage, our method 3 could be implemented. In the case of particles with low permittivity, particles protruding at the surface can be positioned using the topography data. Hence, protrusions with similar dimensions can be compared between the selected specimens, and method 3 can allow the range of possible interphase thickness and permittivity values to be determined. Similarly, when using particles with high permittivity, this approach might allow for the detection of particles in the depth of the material by EFM measurements and also using a statistical approach. Thus, comparing signals between various materials can provide information on the interphase. A correlation with other scanning probe microscopy methods would be also beneficial, such as the nanomechanical techniques that are adapted to similar types of materials with complex geometry and constituents.

## Conclusion

This work demonstrates the accurate detection of interphases in nanocomposites using three EFM-based experimental protocols employing reference samples. As EFM signals represent the synergy of several parameters, the interpretation of the results is not straightforward for the analysis of interfacial effects in nanocomposites. For this reason, in this study, EFM measurements were performed using reference samples with relatively controlled and known properties whereby the samples were prepared to electrostatically model a nanodielectric material with an interphase. In these samples, three types of dielectric materials, each with a specific dielectric permittivity, were assembled in the form of sub-micrometer particles covered by two thin shells that represent the interphase and the matrix in the “real” systems. The study of the signals above the central region of the particles at constant tip–sample distance confirmed that EFM can differentiate between Al_2_O_3_ and SiO_2_ interphase layers of 50 and 100 nm over PS particles (380 nm) and between different types of matrix layers (PVAc, SiO_2_ and Al_2_O_3_). Specifically, three approaches were developed to distinguish the specific signal of the interphase layers based on sample comparison with the following configurations: 1) nanodielectrics (or nanodielectric regions) with and without the upper matrix layer; 2) nanodielectrics with and without interphase, and constant upper matrix thickness, and 3) nanodielectrics with and without interphase, with constant total particle coverage. The quantification of the dielectric permittivity of the used materials, within all types of association, was possible by comparing experimental data and numerical simulations. This paper also discussed the possibility of using the developed approaches to distinguish the composition and dielectric properties of “unknown” interphases in “real” nanocomposites with 3D inclusions, as well as in other types of heterogeneous nanometric materials, with reduced ambiguity about the real origin of the measured signals.

## Experimental

### Samples

The materials were specifically designed and prepared for this study to produce an electrostatic model of a nanodielectric (particle + interphase + matrix). They were based on spherical PS particles deposited on a metallic substrate. Al_2_O_3_, SiO_2_ and PVAc were used to cover the particles and to mimic either the interphase layer or the matrix layer. The PS particle diameter was about 380 nm and the shell thickness was 50 or 100 nm.

### Polystyrene deposition and diameter monitoring

PS particles (initial diameter = 1 µm) (Sigma-Aldrich, ref: 89904) were deposited on previously metallized silicon substrates (Si-Mat Silicon materials, ref: 1014G1007) using the self-assembly property of PS spheres [49–51]. In this work, a more precise experimental protocol than the one used in our previous work [28] was chosen to deposit the particles by spin coating [52–53]. The initial PS sphere solution was mixed with ethanol (1:1 ratio) and kept in an ultrasound bath for 1 min to ensure homogeneous dispersion. Then, 7 µL of this diluted solution was spread over the whole substrate surface just before spinning. Substrates were previously hydrophilized in O_2_ plasma (50 W, 0.001 mbar) for 2 min. The following program was used for the spinning process: a) 100 rpm for 15 s, b) 500 rpm for 30 s and 2000 rpm for 60 s, all with a ramp of 2000 rpm between steps. Then, the obtained films of PS particles were etched in a plasma reactor with O_2_ as the reactive gas. The samples were inserted in the reactor chamber and pumped to reach a vacuum of approximatively 0.011 mbar. O_2_ was then introduced using a needle valve, and the pressure was equilibrated to 0.6 mbar by adjusting the valve. After the equilibrium pressure was reached, a radio frequency power of 50 W at 0.15 A was applied until the desired diameter (around 380 nm after 16 min etching) was obtained [54].

### Shells

**Alumina thin layers: atomic layer deposition (ALD).** The ALD method was used to grow Al_2_O_3_ layers on the nanoparticles [55–56]. ALD is a thin film deposition technique where the film thickness is precisely controlled at the atomic level [57]. The deposition is based on sequential chemical reactions between gas precursors and the material surface. After each cycle of one precursor, an inert gas is introduced to remove the remaining precursor and the resulting by-products. The deposition conditions were the same as in [28]. The final theoretical configuration of the samples was similar to that of the PS + Al_2_O_3_ sample depicted in Figure 1.

**Polyvinyl acetate thin layers: spin coating.** Spin-coating was used to deposit PVAc films on the surface of particles with or without a previous covering layer. A solution containing 0.25 mg PVAc was mixed with 5 mL of toluene (highly evaporating solvent) and stirred with a magnetic stirrer until no particulate was visible (around 30 min). The spinning program used for thin film deposition was: a) 100 rpm for 15 s, b) 500 rpm for 15 s, and c) 2000 rpm for 60 s, all with a ramp of 2000 rpm.

**Silicon dioxide thin films: plasma sputter deposition (PSD).** Silicon dioxide layers were deposited by plasma sputtering in a Plassys 450S reactor using a high purity SiO_2_ target source. The deposition regime included a preliminary exposure to 100 sccm argon plasma at 50 W for 20 s, while substrates and SiO_2_ target samples were screened. Then, the target shutter was opened, and a pre-sputtering step (15 sccm Ar and 0.8 sccm O_2_ plasma gas at 200 W) was maintained for 30 s. Next, the substrate planetary rotation was launched and shutters opened, thus allowing the deposition of the SiO_2_ film. The film thickness was determined by the exposure time to SiO_2_ sputtering. Different from ALD, here the spherical shape of PS particles might not allow SiO_2_ to completely cover the corners between the particles and substrate, although sputtered SiO_2_ molecules invade the whole deposition chamber. Therefore, the final geometry could slightly differ from the PS + SiO_2_ depiction in Figure 3.

### EFM experiments

The samples were mainly studied with EFM, using a double pass method with AC electrical excitation mode, while detecting the force gradients that vary at the double frequency of the electrical bias [58]. The interaction between the EFM tip and an insulator is the combination of the capacitive force between induced charges on the electrodes due to the capacitance *C* relative to the probed region, and a coulombic force between the local surface charges *q*_s_ (if present) and their image charges on the tip – *q*_s_ [58–59]. The general equation of the electrostatic force *F* that describes these interactions is defined as follows:

[3] $$F\;=\;\frac{1}{2}\frac{\partial C}{\partial z}\Delta V^{2}+\frac{q_{\text{s}}q_{\text{t}}}{4\pi \varepsilon_{0}z^{2}}$$

where *z* is the distance between the tip apex and the sample surface, *q*_t_ is the sum of all charges interacting with the surface static charges *q*_s_, and the total voltage difference Δ*V* is expressed as:

[4] $$\Delta V=V_{\text{DC}}+V_{\text{AC}}\sin(\omega t)+V_{\text{CP}}$$

where *V*_DC_ and *V*_AC_sin(ω*t*) are the DC and AC externally applied voltages, respectively, and *V*_CP_ is the contact potential difference; *q*_t_ is expressed as follows:

[5] $$q_{\text{t}}\;=\;q_{\text{s}}+q_{\text{DC}}+q_{\text{AC}}+q_{\text{CP}}$$

where *q*_DC_ = *CV*_DC_, *q*_AC_ = *CV*_AC_sin(ω*t*) and *q*_CP_ = *CV*_CP_ are the capacitive charges due to

*V*_DC_, *V*_AC_sin(ω*t*) and *V*_CP_, respectively.

Consequently, the development of Equation 2 shows that the force, and hence, the force gradient *G* (Nm^−1^), can both be expressed as the sum of the DC, ω and 2ω components. While the force and force gradient detection methods can provide relatively similar information on the electrical properties of the sample, the force gradient detection method is expected to offer higher lateral resolution and sensitivity [60].

The component of the gradient varying at the double of the electrical potential gradient is the only purely capacitive part without the need of further treatment, and it is described as:

[6] $$G_{2\omega }\;=\;-\frac{1}{4}C^{\prime \prime }V_{\text{AC}}{}^{2}\cos2\omega t$$

EFM measurements were performed under ambient air conditions with a commercial AFM (Bruker, previously Veeco, Enviroscope^TM^). The probe consisted of a metal covered tip (µmasch: HQ:NSC18/Pt) supported by a cantilever electrically connected to a metallic sample holder and biased at an electrical potential.

AC-biased EFM (ω = 100π rad) was employed in the double-pass configuration, while exciting the probe at its first eigenmode *f*_0_ [58]. During the first scan, sample topography was extracted and collected on a first image using the tapping mode. For the second scan, the sensor was lifted by a known distance from the surface, the so-called “lift” distance, and controlled to follow the topography profile acquired during the first scan. An AC voltage was then applied between the probe and the sample holder. Furthermore, the mechanical oscillation amplitude was reduced to stay in the linear regime. The detected electrostatic force gradients reduced the effective spring constant of the probe, *K* (N/m), and therefore, modified its resonance frequency. Consequently, at a constant mechanical working frequency, these electric force gradients tended to modify the signal phase, as deduced from the resolution of the tip motion equation [58]. In our experiments, the resonance frequency shifts Δ*f*_0_ were extracted during the second scan by maintaining the phase shift constant throughout the modification of the cantilever exciting frequency.

In the linear regime, Δ*f*_0_ and *G* are related by the following equation [58]:

[7] $$\Delta f_{0}≅-\left(f_{0}/2K\right)\times G$$

Basically, the *G*_2ω_ component was measured using an access module with a lock-in amplifier (EG&G Instruments – Model 5302), locked at the double frequency of the AC-electrical excitation and an arbitrary function generator (Sony Tektronic – AFG310). A custom-made switching device was used to extract the 2ω component from the signal obtained during the second pass. A 5 V AC-voltage with 200π rad/s pulsation was fixed for all EFM measurements.

The results are presented either as frequency shifts (2ω − Δ*f*_0_), or as the EFM parabolic coefficient α_2ω_ (Hz/V^2^), where:

[1] $$\alpha \;\left(2\omega \right)=\;\Delta f_{0}\;\left(2\omega \right)/V^{2}$$

### Numerical simulations

Numerical simulations were obtained using the Comsol^®^ Multiphysics software to corroborate the experimental results and to quantify the permittivity of the sample components.

To model the force acting on the EFM probe, the AC–DC module (electrostatics physics interface) of the software was used [61]. The probe was modeled according to the geometry of the standard EFM tips (i.e., as a solid truncated cone of height *H* = 10 µm and half-angle θ = 10° with a spherical apex of radius *R*_0_ at its end)*,* calibrated for each new tip [28,62–63]. The geometry of each sample was implemented according to the theoretically expected geometry on the basis of the preparation methods (see Figure 1) but it was also verified using the AFM topography results [63].

The thickness of the deposited shells was monitored by characterizing reference samples for each film. However, these samples were prepared over bare substrates. Ellipsometry, electron microscopy and profilometry were used to verify the agreement between the theoretical and the actual Al_2_O_3_, SiO_2,_ and PVAc thickness [38].

2D axisymmetric dimensions were used in accordance with model symmetry when measuring the force at the top of the particle/interphase assembly. The probe was biased at 5 V in DC, while the substrate was grounded. Only the *z* component of the electrostatic force was studied, like in previous finite-element EFM models [64].

**Figure 11:**
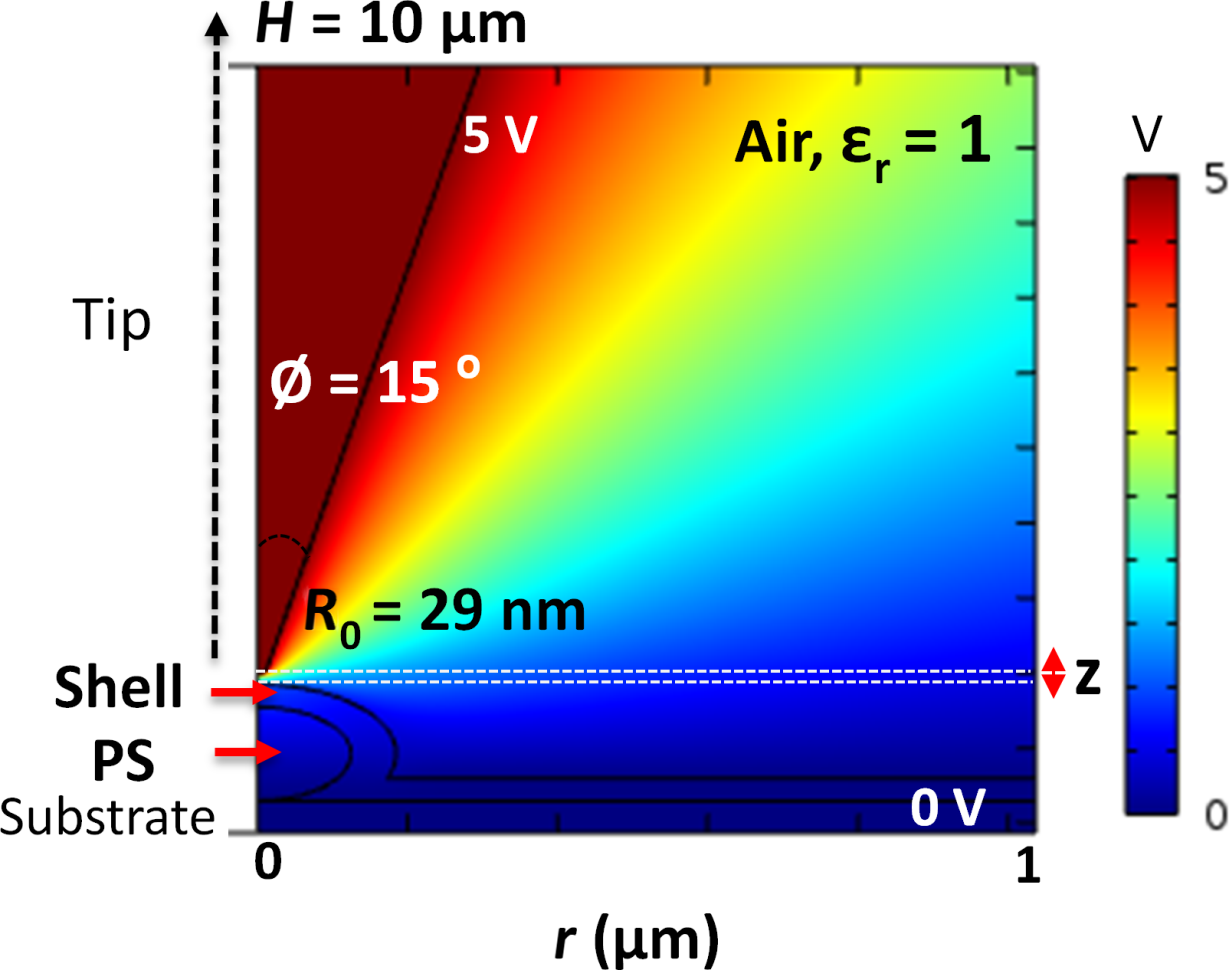
Typical simulation of the electric field map obtained with a 2D axisymmetric model of the EFM tip and the PS + Al_2_O_3_ sample as a substrate.

In our simulations, the purely capacitive DC signal (perfect insulators) was computed (Figure 11). Then, to correlate AC measurements with the simulations, the amplitude of the demodulated 2ω-frequency shift was measured during the experiments. This AC-2ω signal is equal to the half of the DC signal (please refer to the theoretical equations of the DC versus AC-2ω components in [58,65]). Hence, the DC simulation results were divided by a factor of 2 to obtain the corresponding AC-2ω simulation values and compare them to the experimental AC-2ω values.
